# Supplementary material for: Subcellular three-dimensional imaging deep through multicellular thick samples by structured illumination microscopy and adaptive optics
Source: Nat Commun. 2021 May 25;12:3148. doi: 10.1038/s41467-021-23449-6 (PMC8149693; doi:10.1038/s41467-021-23449-6)
Supplement: Supplementary file 1 — Supplementary Information [file 41467_2021_23449_MOESM1_ESM.pdf]

Subcellular three-dimensional imaging deep through multicellular thick samples by structured illumination microscopy and adaptive optics

Supplementary Information

By Ruizhe Lin, Edward T. Kipreos, Jie Zhu, Chang Hyun Khang, and Peter Kner

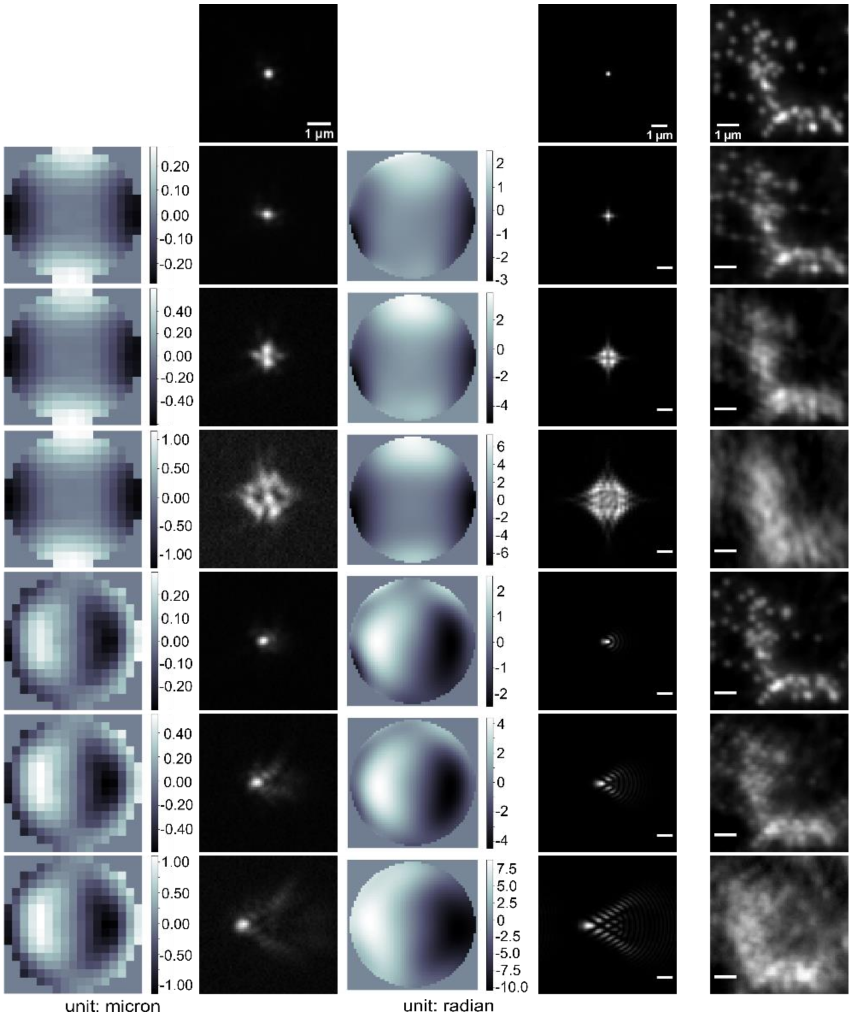

Supplementary Figure 1. We experimentally tested the effect of optical aberrations on the imaging system in order to calibrate the system response to the aberrations with different amplitudes. Below are experimental and theoretical point spread functions (PSF) and images of fluorescent beads (diameter=200 nm) for different Zernike modes and aberration amplitudes. We manually applied three different amplitudes (0.2 μm, 0.4 μm, and 0.8 μm) for two types of Zernike modes (astigmatism and coma) onto the deformable mirror (DM) and took images of single fluorescent beads (diameter=200 nm), as shown in the second column. We measured the wavefront shapes on the DM using the Shack Hartmann Wavefront Sensor (SHWFS), as shown in the first column, and conducted phase retrieval on the aberrated images of the single bead to extract the wavefront phase in the pupil plane, as shown in the third column. Note that the unit of the phase in the measured wavefronts is microns, while the units of the phase retrieval results are radians. Using $k=2\pi/\lambda$ to convert from microns to raidans, for $\lambda=0.515$ μm, 1 micron corresponds to 12.2 radians. We then used the measured wavefront as the pupil function to generate the theoretical PSF, as shown in the fourth column. We also imaged multiple fluorescent beads, as shown in the last column, to associate the spatial images with corresponding wavefront aberrations. The first row is the aberration free case of each column. As Fig. S1 shows, the experimental results generally agree with the theoretical simulations in the extent of image distortions. And we can see that the aberrant wavefronts with peak-to-valley values of ~1.0 micron are already large enough to completely ruin the images, resulting into totally unidentifiable objects.

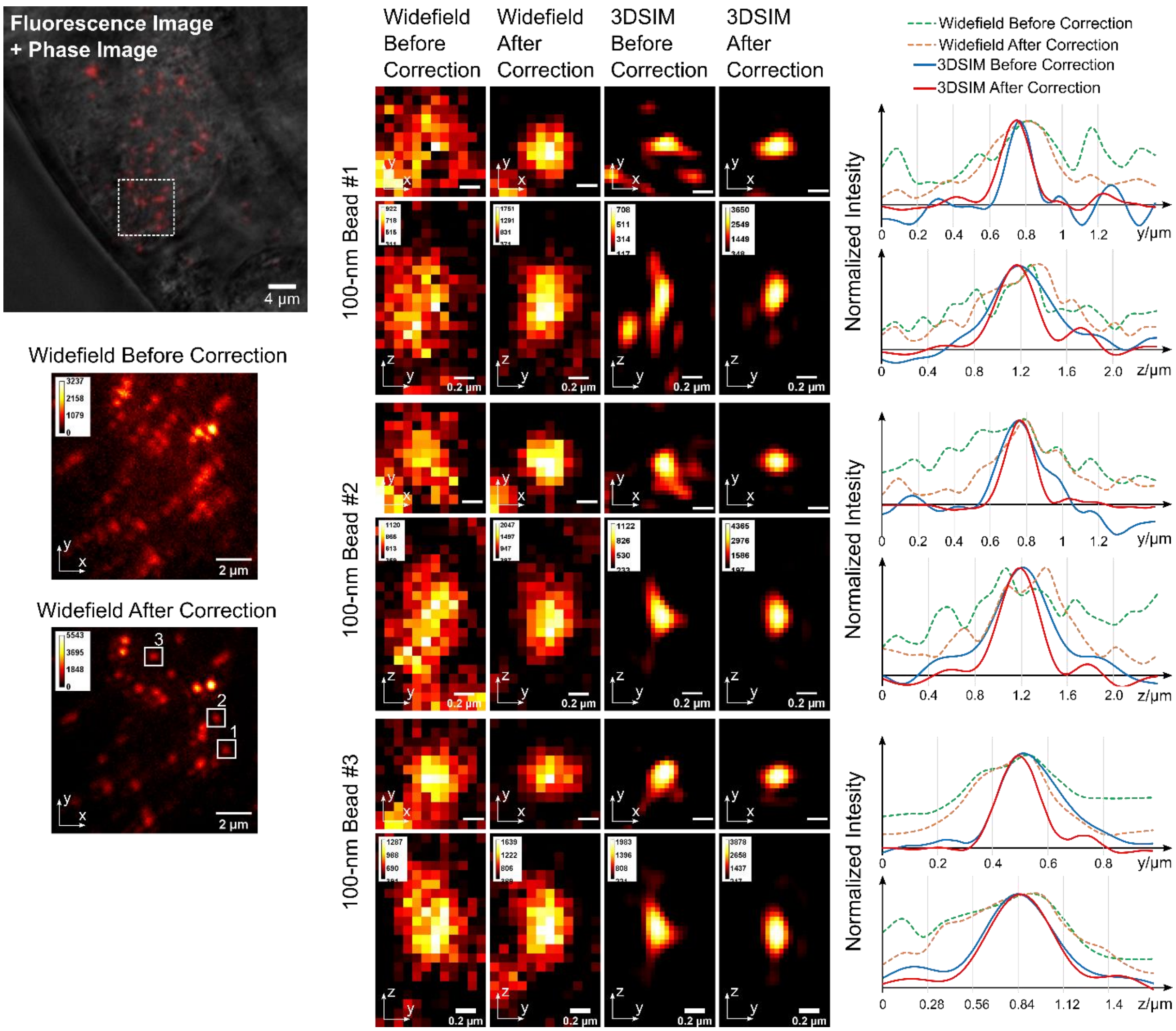

Supplementary Figure 2. The three-dimensional zoomed-in views of three dark-red 100-nm fluorescent beads from Fig. 4 (beads under worm), as well as the intensity profile plotted across the center of the bead along the Y and Z axes. The full width at half maximum (FWHM) of the Gaussian curve fits of the intensity profile can be seen in Supplementary Table 1 below.

Supplementary Table 1. FWHM of images of three100-nm beads (unit: μm)

	Bead #1		Bead #2		Bead #3		Simulation	
	XY	Z	XY	Z	XY	Z	XY	Z
Widefield Before Correction	1.2312	1.5701	0.9686	1.5112	0.711	1.0557	0.2375	0.8680
Widefield After Correction	0.5366	0.8393	0.5238	0.8007	0.3546	0.7465		
3DSIM Before Correction	0.1247	0.8562	0.2104	0.8252	0.2494	0.7366	0.1257	0.3926
3DSIM After Correction	0.1580	0.5524	0.1491	0.5389	0.1446	0.6360		

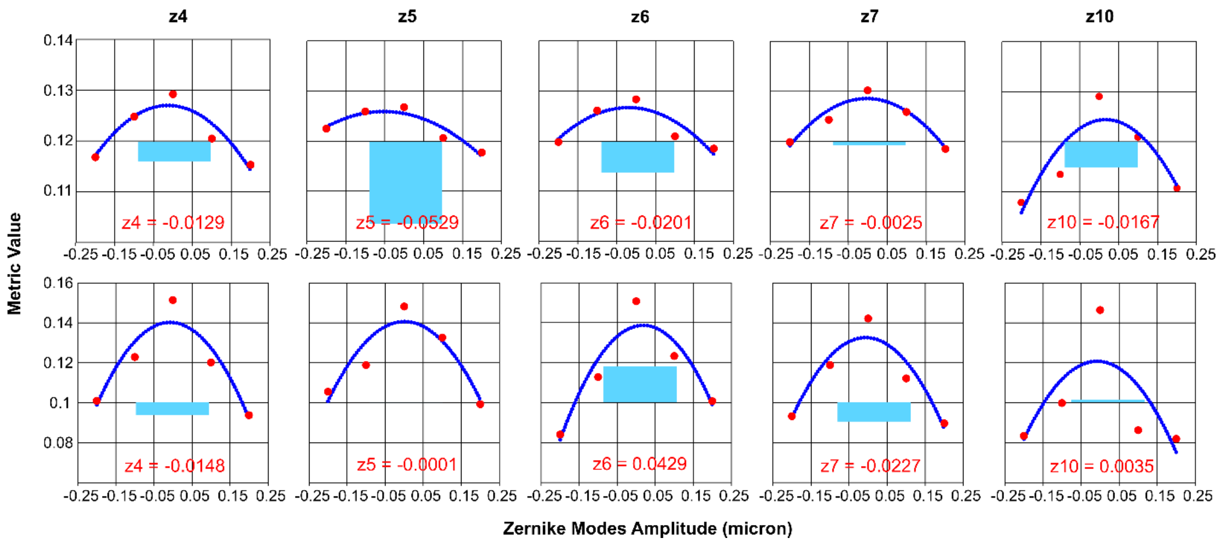

Supplementary Figure 3. The metric values and the quadratic curve fitting in sensorless AO correction. The top row is the result from imaging the rice fungus (Fig. 5), the bottom row is the results from imaging the *C. elegans* axon (Fig. 6).

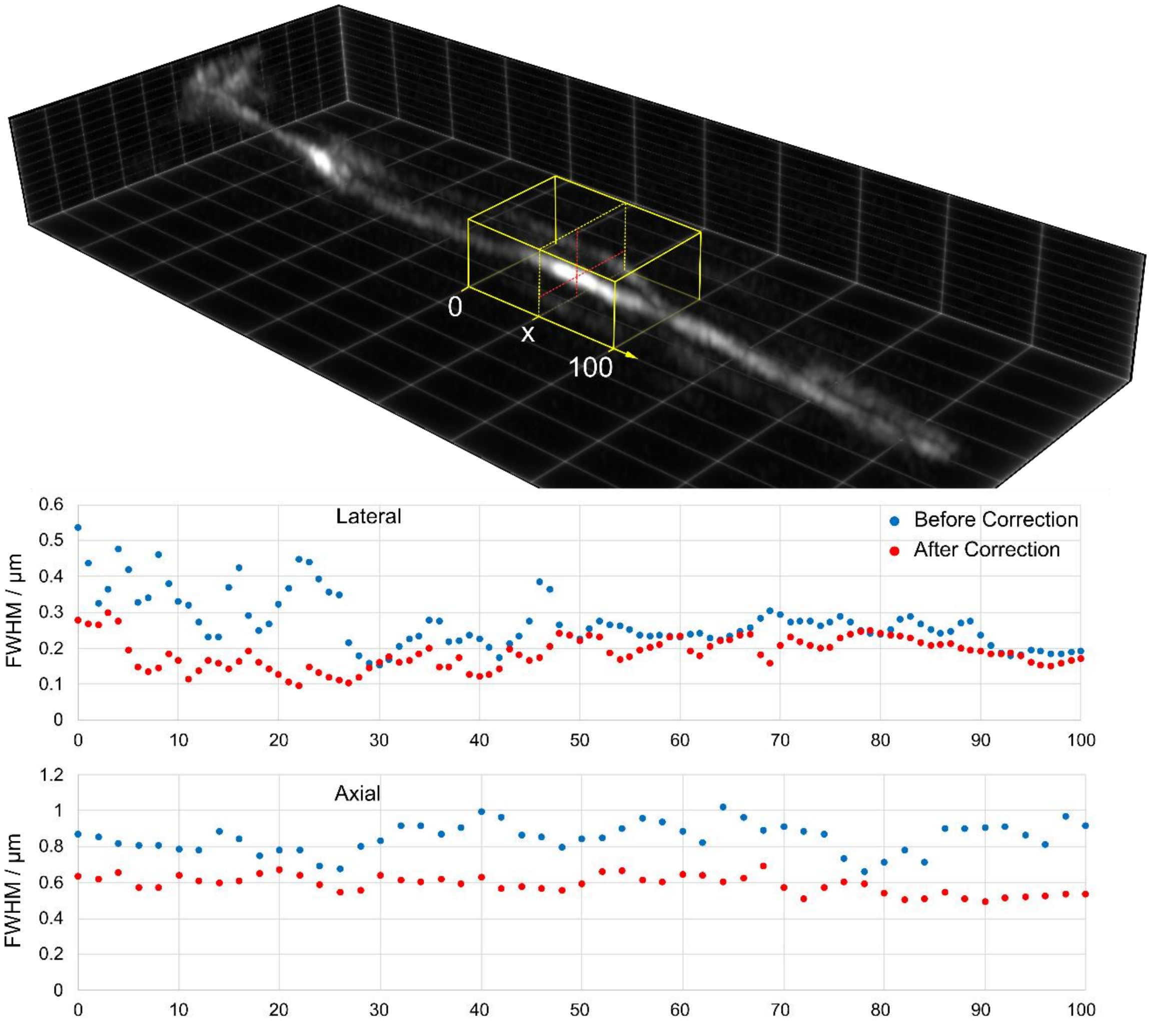

Supplementary Figure 4. The plots of the FWHM values of the Gaussian curve fits of the *C. elegans* axon profile along a length of 100 pixels (1 pixel = 44.5 nm). This is a 3D visualization of the GFP-labelled RIC interneurons in Fig. 6. The lateral and axial FWHM are calculated for every cross-sectional frame (as illustrated by the yellow dashed line square) in the yellow box. The axial FWHM is calculated for every other cross-sectional frame. From the plotted dots, we can see a consistent improvement of FWHM in both the lateral and axial directions.

Supplementary Table 2. SNR Results*

AO Method	Sample	Exposure Time (s)	EMCCD Gain	SNR				Number of images taken for AO correction	Number of images taken for 3D-SIM
				Widefield		3D-SIM			
				Before AO	After AO	Before AO	After AO		
Widefield Sensorless	Actin (Fig. 3)	0.1	150	5.37	6.34	20.63	33.48	50	390
	Beads (Fig. 4)	0.1	200	9.16	16.35	19.79	33.73	50	315
	ER (Fig. 5)	0.1	150	4.90	5.29	8.74	46.06	25	390
	Axon (Fig. 6)	0.05	100	8.85	17.16	16.97	33.78	25	315
Confocal Sensorless	AJs (Fig. 7)	0.1	150	4.88	5.98	4.63	4.75	65	390
Direct Wavefront Sensing**	ER (Fig. 8)	0.1	160	4.96	5.04	6.10	13.91	5	315
	AJs (Fig. 9)	0.1	50	5.07	5.51	4.74	34.40	5	450
	Axon (Fig. 10)	0.1	100	9.86	13.95	33.53	57.12	3	420

* Laser Intensity is ~ 6 W/cm^2

** Wavefront sensor exposure time is 0.25s

Supplementary Table 3. The lateral resolutions of widefield images measured by decorrelation analysis [1]

Widefield		Before Correction	After Correction
Widefield Sensorless	Cell actin	0.533	0.441
	Beads Under *C. elegans*	0.460	0.416
	Fungus ER	0.580	0.297
	C. elegans Axon	0.721	1.332
Confocal Sensorless	*C. elegans* AJs	0.765	0.543
Confocal Direct Wavefront Sensing	*C. elegans* AJs	0.552	0.529
	Fungus ER	1.917	0.486
	C. elegans Axon	0.681	0.355

(unit: µm)

Supplementary Table 4. Comparison of the FWHM values of the Gaussian curve fits of the intensity profiles of fungus ER before and after sensorless and confocal direct wavefront sensing AO corrections

3D-SIM		Sensorless (Fig. 5)		Confocal Direct Wavefront Sensing (Fig. 9)	
		Before Correction	After Correction	Before Correction	After Correction
Lateral (XY)	1	0.372	0.307	0.234	0.151
	2	0.438	0.234	0.485	0.223
	3	0.274	0.198	0.311	0.238
Axial (Z)	1	0.887	0.741	0.567	0.524
	2	1.056	0.803	0.708	0.449
	3	0.971	0.947	0.645	0.445

(unit: µm)

Supplementary Table 5. Comparison of the FWHM values of the Gaussian curve fits of the intensity profiles before and after confocal direct wavefront sensing AO corrections

3D-SIM		*C. elegans* AJs (Fig. 8)		*C. elegans* Axons (Fig. 10)	
		Before Correction	After Correction	Before Correction	After Correction
Lateral (XY)	1	0.484	0.344	0.338	0.269
	2	0.878	0.389	0.400	0.335
	3	1.240	0.408	0.540	0.412
Axial (Z)	1	0.955	0.660	0.732	0.477
	2	0.977	0.845	1.034	0.832
	3	0.770	0.641	1.659	0.736

(unit: µm)

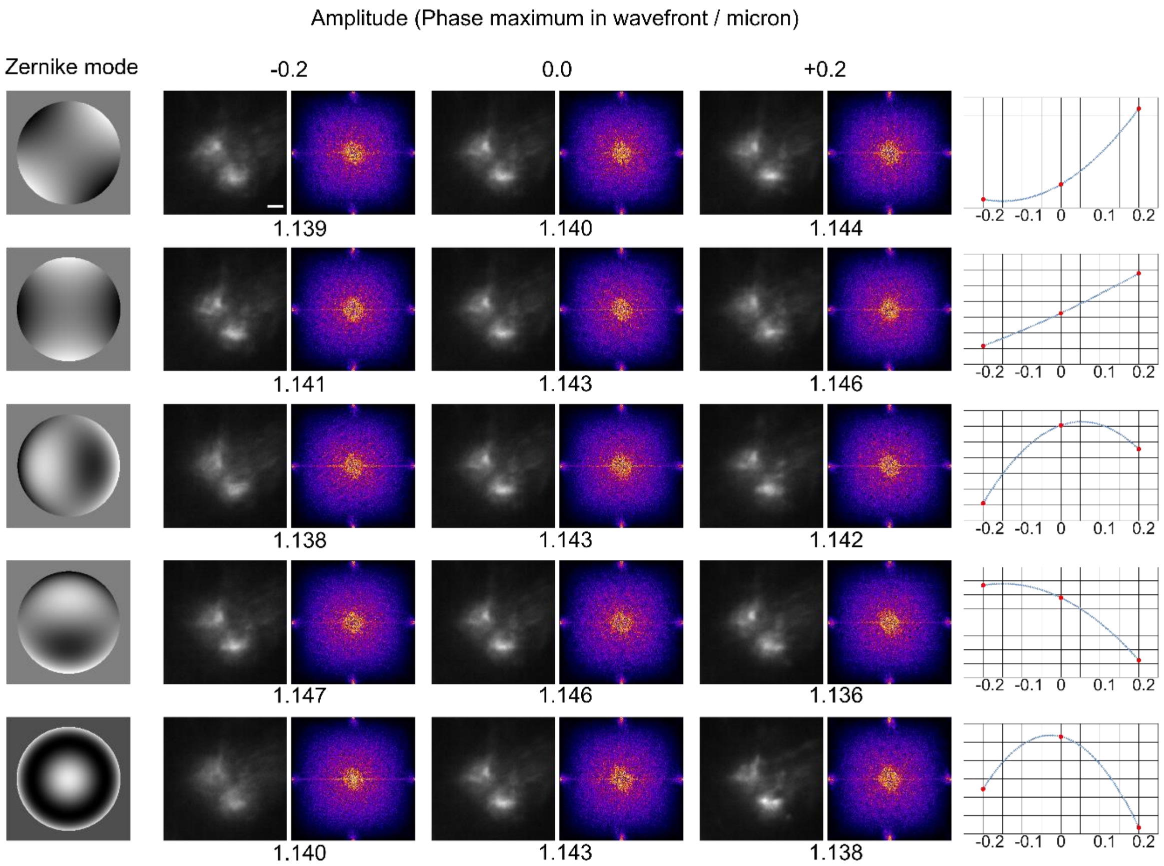

Supplementary Figure 5. The sensorless AO iterations based on the widefield image of the *C. elegans* expressing the *ajm-1*::GFP reporter in adherens junctions in the posterior bulb of the pharynx. The metric values of each image are calculated with the high and low frequency bandwidth being tuned optimally after experiments. It is obvious that the sensorless method failed to find the optimal amplitudes for astigmatism. The frequency-based metric function performs poorly in this scenario, where the object is so blurry that the frequency spectrum is quite uniform and lacks useful information.

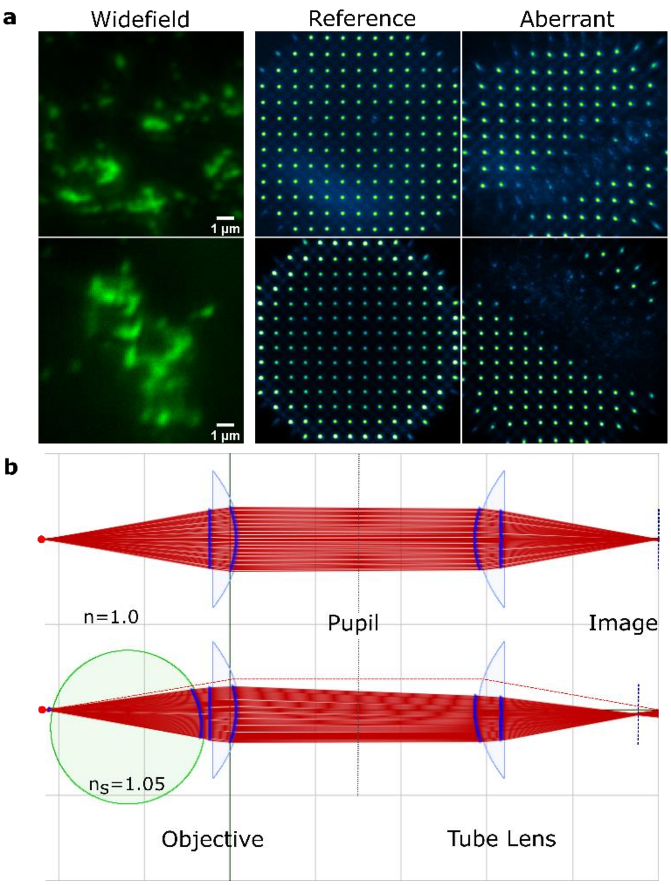

Supplementary Figure 6. a. Widefield images of the fluorescent beads under the *C. elegans* (left column) and their corresponding focal spot arrays on the SHWFS (right column). We can see a lot of missing spots due to the large aberrations induced by the worm body, preventing the wavefront reconstruction from working. As a result, the direct wavefront sensing method cannot correct the optical aberrations on this sample, while the sensorless method gives a decent result (see Fig. 4). As we tested in experiments, the missing spots cannot be recovered after running sensorless AO, which implies that this is a scenario that direct wavefront sensing is not able to handle. **b.** The missing spots in the Shack-Hartmann image are due to strong refraction in the sample which changes the amplitude profile in the back pupil plane. Under the normal assumption of weak phase variations, each ray accumulates a phase change but is not bent in the sample, and the back pupil of the objective is filled uniformly, but this is not always the case in biological samples. This is illustrated by the simulation in which the body of the organism focuses the fluorescence from a point source before it reaches the objective, resulting in the back pupil plane being underfilled. Here, the worm body is simulated by a round object with the refractive index 5% higher than the environment, as the biological components in the tissue have the refractive indices ranging from 1.36-1.49 [2], which is generally ~5% higher than the water with refractive index of 1.33.

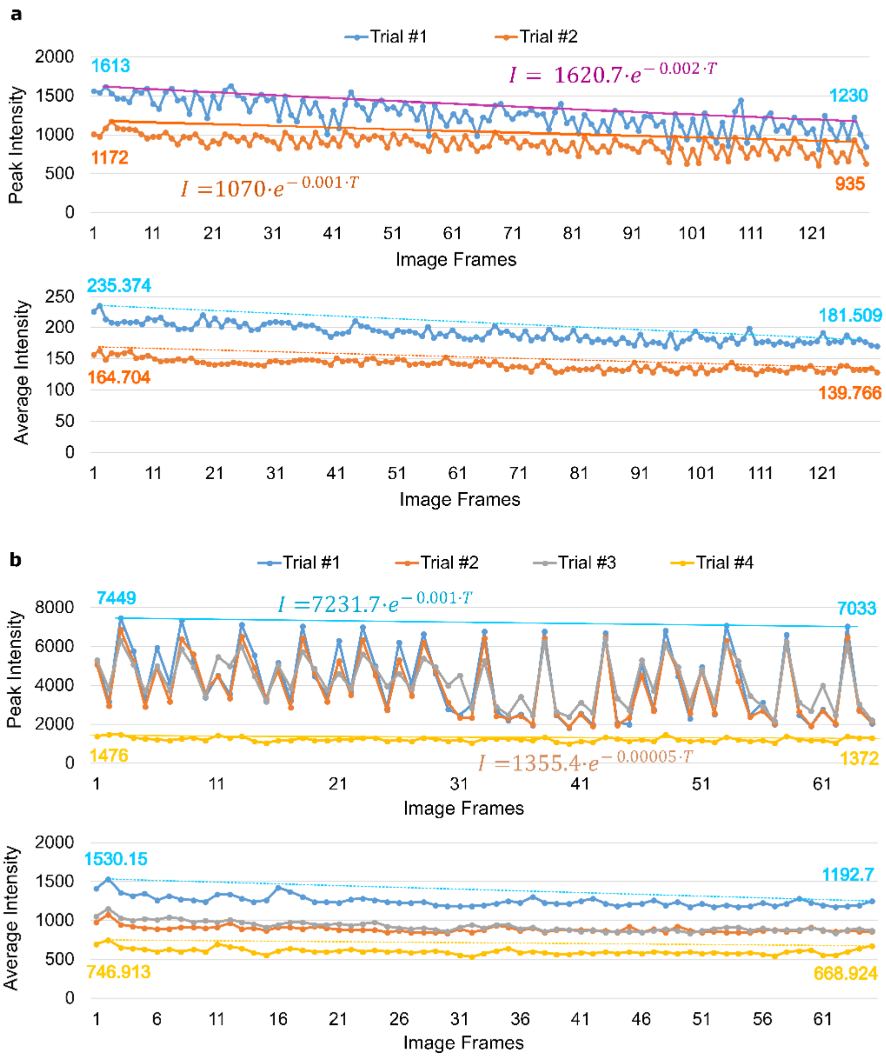

Supplementary Figure 7. Plots of the peak and average fluorescence intensities over image frames in a photobleaching test with extended sensorless AO iterations: **a.** GFP in live *C. elegans*. **b.** Alexa Fluor 647 dye in fixed cells. The dots are experimentally measured signal intensities. The lines are the fitted exponential decay functions that indicate the intensity trend in the tests of the photobleaching effect during the extended sensorless AO iterations. The fluctuation of the peak and average intensities is due to different trial aberrations applied with each image frame. The actual numbers of image frames taken for sensorless AO corrections are listed in Supplementary Table 2.

For a typical number of sensorless AO iterations, 25, the amount of photobleaching is ~ 2.2% to 4.4%. For an AO-3DSIM stack with 390 raw images, the amount of photobleaching is ~ 30% to 50%.

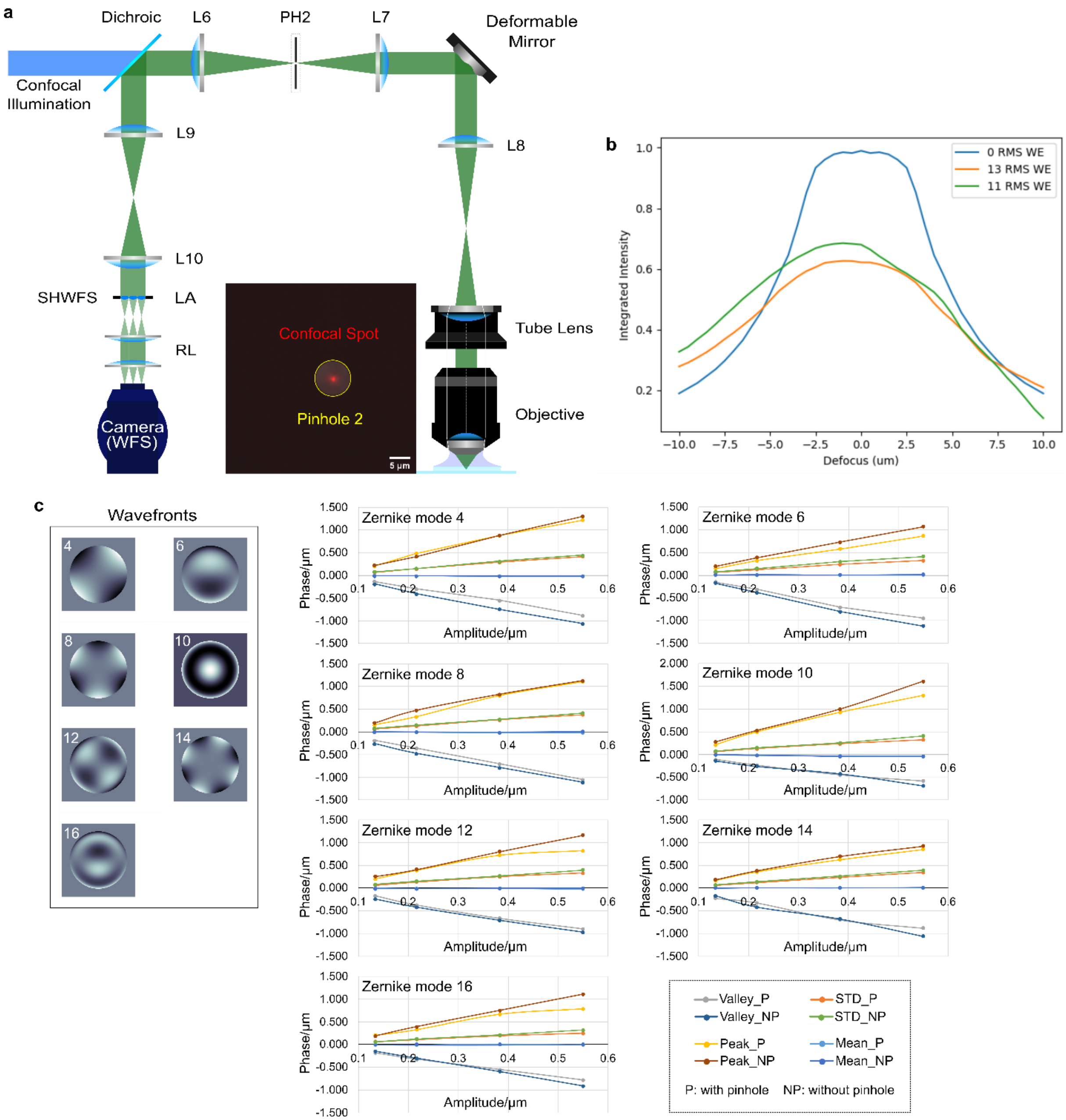

Supplementary Figure 8. a. The experimental setup for testing the performance of the Shack-Hartmann Wavefront Sensors (SHWFS) using the confocal spot as the guide star. The inset shows an image overlay of the pinhole, PH2, (grey, yellow outline) and the confocal spot (red). The pinhole, PH2, diameter 500µm, is placed at the conjugated image plane after the deformable mirror (DM) and corresponds to a size of 8.3 µm at the sample. Artificial aberrations are simulated by applying the Zernike modes, as shown in the square, onto the DM with different amplitudes. **b.** Simulation of integrated intensity as a function of defocus for a point source, including the effect of the pinhole. The pinhole only effects the signal for large aberrations. The RMS wavefront error is in radians. c. Plots of the peak, valley, standard deviation, and mean values of the wavefronts measured by the SHWFS with and without the pinhole. The results show similar wavefront measurements with and without the pinhole. Deviations start to appear for RMS amplitudes greater than ~0.5 µm.

Following the analysis in in sec. 2.7 of Tao et al.[3], a pinhole of size λ/d_{sub} will attenuate spatial frequencies above $1/2d_{\mathrm{sub}}$. For our wavefront sensor, the lenslet pitch is 150 microns. With $\lambda = 509\,nm,$ this corresponds to a pinhole size of 3.4 milliradians. At the position of PH2, this corresponds to 288 microns. Therefore, a pinhole of 500 microns will not limit the measurement of frequencies that can be measured by our wavefront sensor.

The size of the pinhole image on the deformable mirror is an Airy Disk with central lobe diameter of 0.6 mm, much less than the actuator pitch of 1.5mm. This indicates that the pinhole size is does not affect the measurement of the wavefront at the scale that can be corrected by the DM. The pinhole and the deformable mirror are respectively in the front and back focal planes of a 250mm focal length achromat. The deformable mirror actuator pitch is 1.5mm and, for small actuator throw, the DM can be thought of as a grating with grating wavelength 3mm. For $\lambda = 509\,nm$, the DM will deflect light at a maximum angle of ~ 2e-4 which corresponds to a deflection of ~ 50 microns at the pinhole location. Therefore, for small wavefront errors, the pinhole has no effect. For larger wavefront errors, the pinhole will begin to affect the results. This is because the frequency content of the beam will increase as the wavefront error increases – more terms must be kept in the expansion of $\exp(-j\varphi(\bar{r}))$ as $\varphi(\bar{r})$ increases. As we show in Fig. S8(b), the pinhole in our setup does not begin to affect the wavefront measurement until the RMS amplitude exceeds ~0.5μm.

Reference

1. Descloux A, Grußmayer KS, Radenovic A. Parameter-free image resolution estimation based on decorrelation analysis. *Nature Methods* **16**, 918-924 (2019).

2. BASHKATOV AN, GENINA EA, TUCHIN VV. OPTICAL PROPERTIES OF SKIN, SUBCUTANEOUS, AND MUSCLE TISSUES: A REVIEW. *Journal of Innovative Optical Health Sciences* **04**, 9-38 (2011).

3. Tao X, *et al.* Live imaging using adaptive optics with fluorescent protein guide-stars. *Opt Express* **20**, 15969-15982 (2012).
