# Supplementary material for: Subcellular three-dimensional imaging deep through multicellular thick samples by structured illumination microscopy and adaptive optics
Source: Nat Commun. 2021 May 25;12:3148. doi: 10.1038/s41467-021-23449-6 (PMC8149693; doi:10.1038/s41467-021-23449-6)
Supplement: Supplementary file 4 — Reporting Summary [file 41467_2021_23449_MOESM4_ESM.pdf]

nature research

Corresponding author(s): Peter Kner

Last updated by author(s): Apr 21, 2021

Reporting Summary

Nature Research wishes to improve the reproducibility of the work that we publish. This form provides structure for consistency and transparency in reporting. For further information on Nature Research policies, see our Editorial Policies and the Editorial Policy Checklist.

Statistics

For all statistical analyses, confirm that the following items are present in the figure legend, table legend, main text, or Methods section.

n/a	Confirmed	
☐	☒	The exact sample size (*n*) for each experimental group/condition, given as a discrete number and unit of measurement
☒	☐	A statement on whether measurements were taken from distinct samples or whether the same sample was measured repeatedly
☒	☐	The statistical test(s) used AND whether they are one- or two-sided<br>*Only common tests should be described solely by name; describe more complex techniques in the Methods section.*
☒	☐	A description of all covariates tested
☒	☐	A description of any assumptions or corrections, such as tests of normality and adjustment for multiple comparisons
☐	☒	A full description of the statistical parameters including central tendency (e.g. means) or other basic estimates (e.g. regression coefficient) AND variation (e.g. standard deviation) or associated estimates of uncertainty (e.g. confidence intervals)
☒	☐	For null hypothesis testing, the test statistic (e.g. *F*, *t*, *r*) with confidence intervals, effect sizes, degrees of freedom and *P* value noted<br>*Give P values as exact values whenever suitable.*
☒	☐	For Bayesian analysis, information on the choice of priors and Markov chain Monte Carlo settings
☒	☐	For hierarchical and complex designs, identification of the appropriate level for tests and full reporting of outcomes
☒	☐	Estimates of effect sizes (e.g. Cohen's *d*, Pearson's *r*), indicating how they were calculated

Our web collection on statistics for biologists contains articles on many of the points above.

Software and code

Policy information about availability of computer code

Data collection	Data was collected using custom code written in python. This code controlled the camera, spatial light modulator, lasers, and all other computer controllable pieces of the microscope. The software saved the raw data as three-dimensional tif files along with an accompanying text file that included relevant metadata. The software is available upon request and can be downloaded from github.com/Knerlab (DOI:10.5281/zenodo.4690769). All the softwares use Python 3.6.<br>We used the Python package of scikit-image (version:0.16.2) for extracting the object in signal-to-noise ration calculations.<br>We used the ImageJ plugin Image Decorrelation Analysis (Version 1.1.7) for evaluating the image resolutions.<br>We used the ImageJ plugin ClearVolume for generating the supplementary movies.
Data analysis	The superresolution images were reconstructed from the raw data (the image stacks saved by the data collection software) using custom software written in python. The software is available upon request and can be downloaded from github.com/Knerlab (DOI:10.5281/zenodo.4690773). The software uses Python 3.6.

For manuscripts utilizing custom algorithms or software that are central to the research but not yet described in published literature, software must be made available to editors and reviewers. We strongly encourage code deposition in a community repository (e.g. GitHub). See the Nature Research guidelines for submitting code & software for further information.

Data

Policy information about availability of data

All manuscripts must include a data availability statement. This statement should provide the following information, where applicable:

- Accession codes, unique identifiers, or web links for publicly available datasets
- A list of figures that have associated raw data
- A description of any restrictions on data availability

Source data are provided with this paper. The image data (raw data, reconstructed images, and image metadata) are available on https://www.ebi.ac.uk/biostudies/studies/S-BSST629.

Field-specific reporting

Please select the one below that is the best fit for your research. If you are not sure, read the appropriate sections before making your selection.

☒ Life sciences ☐ Behavioural & social sciences ☐ Ecological, evolutionary & environmental sciences

For a reference copy of the document with all sections, see nature.com/documents/nr-reporting-summary-flat.pdf

Life sciences study design

All studies must disclose on these points even when the disclosure is negative.

Sample size	For all the structures imaged in the manuscript (alpha-TN4 actin, C. elegans RIC interneuron, C. elegans adherens junctions, and M. oryzae endoplasmic reticulum) at least three samples were imaged to confirm that the images improved with the application of structured illumination microscopy and adaptive optics. For the measurements of bead images in Fig. 4, five beads were measured so that we saw a statistical difference comparing most cases (with and without structured illumination and with and without adaptive optics).
Data exclusions	No data were excluded.
Replication	For every figure in the manuscript, we have imaged more than three samples and to confirm consistent results.
Randomization	Randomization was not relevant to this study. Each sample was measured both with and without structured illumination and with and without adaptive optics.
Blinding	Blinding was not relevant to this study. Each sample was measured under all four conditions, and the microscopist had to determine and apply the imaging approach for each measurement.

Reporting for specific materials, systems and methods

We require information from authors about some types of materials, experimental systems and methods used in many studies. Here, indicate whether each material, system or method listed is relevant to your study. If you are not sure if a list item applies to your research, read the appropriate section before selecting a response.

Materials & experimental systems

n/a	Involved in the study	
☒	☐	Antibodies
☐	☒	Eukaryotic cell lines
☒	☐	Palaeontology and archaeology
☐	☒	Animals and other organisms
☒	☐	Human research participants
☒	☐	Clinical data
☒	☐	Dual use research of concern

Methods

n/a	Involved in the study	
☒	☐	ChIP-seq
☒	☐	Flow cytometry
☒	☐	MRI-based neuroimaging

Eukaryotic cell lines

Policy information about cell lines

Cell line source(s)	Alpha-TN4 cell line original source: Dr. Paul Russell's laboratory (NIH)<br>Yamada, T., Nakamura, T.,Westphal, H., Russell, P., 1990. Synthesis of alpha- crystallin by a cell line derived from the lens of a transgenic animal. Curr. Eye Res. 9, 31e37.<br>Magnaporthe oryzae strain CKF4019 was newly generated in this study by genetically transforming M. oryzae wild-type strain O-137 with a green fluorescent protein construct.
Authentication	The cell line was not authenticated
Mycoplasma contamination	Alpha-TN4 cells are routinely assessed. Negative as measured by PCR (UniversalMycoplasma Detection Kit (ATCC® 30-1012K™))<br>M. oryzae strain was not tested for mycoplasma contamination.
Commonly misidentified lines<br>(See ICLAC register)	No commonly misidentified lines have been identified in the ICLAC register.

Animals and other organisms

Policy information about studies involving animals; ARRIVE guidelines recommended for reporting animal research

Laboratory animals	Caenorhabditis elegans<br><br>Strains: MT9971<br>Genotype: nIs107 [tbh-1::GFP + lin-15(+)] III.<br>Age: Adult<br>Sex: Hermaphrodites<br><br>Strains: BR2958<br>Genotype: jcIs1 [ajm-1::GFP + unc-29(+) + rol-6(su1006)] IV. ngEx1[ceh-16::GFP].<br>Age: Young Adults<br>Sex: Hermaphrodites
Wild animals	This study does not involve wild animals.
Field-collected samples	This study does not involve samples collected from the field.
Ethics oversight	No ethical approval or guidance is required because animal welfare guidance does not cover low invertebrates, i.e. nematodes.

Note that full information on the approval of the study protocol must also be provided in the manuscript.
