# Supplementary material for: Subcellular three-dimensional imaging deep through multicellular thick samples by structured illumination microscopy and adaptive optics
Source: Nat Commun. 2021 May 25;12:3148. doi: 10.1038/s41467-021-23449-6 (PMC8149693; doi:10.1038/s41467-021-23449-6)
Supplement: Supplementary file 5 — Description of Additional Supplementary Files [file 41467_2021_23449_MOESM5_ESM.pdf]

Title: Supplementary Movie 1.
Description: This movie shows the data from Figure 10, TBH1::GFP expressed in an RIC interneuron in live C. elegans. 3D-SIM with system correction. The 3D visualization is generated using ClearVolume. In the video with system correction, fluorescence can be seen above the neuron from the cell body. This results from the lower SNR and degraded axial resolution without sample correction and cannot be seen in the video with sample correction because the cell body is above the imaged volume.

Title: Supplementary Movie 2.
Description: This movie shows the data from Figure 10, TBH1::GFP expressed in an RIC interneuron in live C. elegans. 3D-SIM with sample correction.
